# A Treatise on Common Forms of Functional Nervous Diseases

**Published:** 1880-10

**Authors:** 


A Treatise on Common Forms of Functional Nervous Diseases. By L. Putzel, M.D., Physician to the Clinic for Nervous Diseases, Bellevue Hospital, Out Door Department, etc., etc. Wm. Wood & Co., New York. 1880.

As a work on the multitude of function nervous diseases the above is calculated to give much information and profit. It goes far toward making the series of "Wood's Library of Standard Medical Authors" complete and all that could be desired.

